# Comparative 2D-DIGE Proteomic Analysis of Bovine Mammary Epithelial Cells during Lactation Reveals Protein Signatures for Lactation Persistency and Milk Yield

**DOI:** 10.1371/journal.pone.0102515

**Published:** 2014-08-11

**Authors:** Jagadeesh Janjanam, Surender Singh, Manoj K. Jena, Nishant Varshney, Srujana Kola, Sudarshan Kumar, Jai K. Kaushik, Sunita Grover, Ajay K. Dang, Manishi Mukesh, B. S. Prakash, Ashok K. Mohanty

**Affiliations:** 1 Animal Biotechnology Center, National Dairy Research Institute, Karnal, India; 2 Department of Biophysics, All India Institute of Medical Sciences, New Delhi, India; 3 Dairy Cattle Physiology Division, National Dairy Research Institute, Karnal, India; 4 National Bureau of Animal Genetic Resources, Karnal, India; University of Edinburgh, United Kingdom

## Abstract

Mammary gland is made up of a branching network of ducts that end with alveoli which surrounds the lumen. These alveolar mammary epithelial cells (MEC) reflect the milk producing ability of farm animals. In this study, we have used 2D-DIGE and mass spectrometry to identify the protein changes in MEC during immediate early, peak and late stages of lactation and also compared differentially expressed proteins in MEC isolated from milk of high and low milk producing cows. We have identified 41 differentially expressed proteins during lactation stages and 22 proteins in high and low milk yielding cows. Bioinformatics analysis showed that a majority of the differentially expressed proteins are associated in metabolic process, catalytic and binding activity. The differentially expressed proteins were mapped to the available biological pathways and networks involved in lactation. The proteins up-regulated during late stage of lactation are associated with NF-κB stress induced signaling pathways and whereas Akt, PI3K and p38/MAPK signaling pathways are associated with high milk production mediated through insulin hormone signaling.

## Introduction

Lactation is an important aspect of mammalian reproductive physiology where the nourishment of the young takes place with copious milk secretion by mammary epithelial cells (MEC) in mammary gland. Mammary gland is made up of a branching network of ducts that end with alveoli. Epithelial cells surround a lumen in each alveolus and these alveolar epithelial cells function by converting nutrients from the blood and transforming them into the molecular components of milk followed by secretion into the lumen [1]. MEC reflects the milk producing ability, so the number and the secretory activity of MECs are very important for milk yield. Lactation is one of the most important phase among the five developmental phases of postnatal mammary gland [2]. The changes in the lactation stages can be distinguished clearly as rate of milk synthesis by function of time postpartum. The biochemical and cytological changes within the mammary epithelium has been linked to the shape of the lactation curve representing the 4 phases. The phase I and II are considered as gain-of function phase (early stage), phase III is at peak in milk synthesis (peak stage) and phase IV is loss-of function phase (late stage) [3], [4]. Stages of lactation have a marked influence on milk yield and resulting milk constituents in dairy animals. In cattle breeds, post calving, milk yield is initiated at relatively high level and reaches to its peak approximately 3 to 6 weeks after parturition. This peak stage is maintained for few weeks which declines gradually. Lactation persistency is an important phenomenon in lactating mother which can be defined as the sustenance of milk yield for the full lactation length in cattle breeds. This is essential for the nursing of the neonates and economics of milk production in farm animals. The milk yield and its constituents fluctuate widely during lactation due to genetic and environmental factors. The pre-peak increase in milk yield is mainly due to the activity and functioning of MEC, whereas the gradual decline in post-peak production is due to the decrease in number of MECs [5]. To establish and maintain proper lactation, the mammary epithelium must be polarized, requiring a three-dimensional architecture with intact tight junctions between adjacent MECs and attachment to the extracellular matrix [6]–[8]. The developmental changes that occur within the mammary gland during lactation stages at molecular level are relatively less analyzed or not clearly understood.

The biological shut-down of milk production called as secretory diminution is the major factor which defines the lactation persistency [9], [10]. The concern of secretory diminution is not only in diary species, but also in humans. It is clear that a significant number of women who choose to breastfeed are incapable of sustaining lactation even up to 6 months, the benchmark supported by both American Pediatrics Association and the “Healthy People 2010” initiative of the United States Department of Health and Human Services [11], [12]. This suggests that it is important to understand the mechanisms of regulation of lactation stages especially the late stage or secretory diminution not only to the dairy industry but also to human health. Although genetic and non-genetic factors such as hormonal status, animal and udder health, stress and nutrition have been reported to be responsible for lactation persistency and milk yield, the expression of various proteins in MECs and their interactions decide whether the milk yield will persist or diminish. Therefore understanding the expression dynamics of various proteins in MECs of lactating mother will reveal some answers to secretory diminution and milk producing ability during lactation

In our previous study, we reported the global proteome of bovine mammary epithelial cells [13] and various groups had reported proteomic studies of farm animal milk proteomics [14], [15]. However there is no report on proteomic changes in mammary epithelial cells with substantial difference in milk producing ability or lactation stages. In the present investigation we report the proteome dynamics of MECs isolated from lactating cows during different stages of lactation i.e. early, peak and late stages and also compared the proteome difference in high and low milk producing cows at peak stage. The lactating cows belonged to Sahiwal breeds which are recognized indigenous milch breeds. We used 2D-DIGE technology for quantitative analysis of differentially expressed proteins that allows detection of minute changes in protein abundance with statistical significance [16]–[19]. The differentially expressed proteins were identified by MALDI-TOF-TOF and LC-MS/MS analysis. The results were further validated by real-time PCR and western blotting. This study provides, for the first time the proteome changes in MECs during different stages of lactation and changes associated with milk synthesis.

## Materials and Methods

### 1. Ethics Statement

Approval of Institute Animal Ethics (IAEC) committee was not required because the experiment did not involve any invasive procedures for animal experiment. The milk samples were collected from the dairy herd of National Dairy Research Institute (NDRI), Karnal which is a public funded research institute under Indian Council of Agricultural Research, Government of India. The milk is routinely collected every day from the lactating animals for routine care and research purpose. The animals were maintained under expert veterinary supervision. NDRI has all the correct permits for the housing and care of animals for scientific purposes vide registration no. 1705/GO/ac/13/CPCSEA 3rd July, 2013 duly approved by Ministry of Environment and Forest, Govt. of India (Web site: http://envfor.nic.in).

### 2. Selection of animals

The animals for the study were taken from the dairy herd of National Dairy Research Institute (Karnal, India). The milk samples were collected from lactating cows belonging to Sahiwal breeds whcih were maintained under expert veterinary supervision. The animals were healthy without incidence of clinical or subclinical mastitis, which were routinely tested by somatic cell counts (SCC) and California Mastitis Test. All the animals selected for these studies were in their 3^rd^ or 4^th^ parity. For the comparative proteomic analysis at different stages of lactations, we selected four animals in each group of immediate early (E, days 15–30 post-parturition), peak (P, days 75–100 post-parturition) and late stage (L, days 210–250 post-parturition) of lactation. Similarly, four animals each of indigenous Sahiwal cows with high yielding (Hy, ∼15 liters/day) and low-yielding (Ly, ∼5 liters/day) breeds and four high-yielding cross bred cows (Karan Fries: KF, 18–22 liters/day) were selected which were at peak stage of their lactation. The animal data was collected from the records maintained at Animal Genetics and Breeding department, National Dairy Research Institute (NDRI), Karnal, India. All the animals were maintained in a controlled environment in animal herd of NDRI till completion of the entire study.

### 3. Isolation of MECs from milk

The MECs were isolated from the milk samples of all the selected animals as described previously [13]. Briefly, two to four litters of fresh milk samples were cooled to 4°C and centrifuged (15 min, 1500× g) in several 250 mL centrifuge bottles at 4°C. Fat layer was removed with spatula and the remaining skim milk was discarded. The cell pellet was washed twice (10 min, 1000× g) with 100 mL of cold PBS and the final pellet was pooled and resuspended in PBS containing 0.1% BSA to give a final cell count of (5×10^6^) cells/mL. MECs were isolated by Immunomagnetic separation technique using Dynabeads (Pan Mouse IgG, Dynal Biotech, Invitrogen) according to the manufacturer's protocol and the methods described previously [13], [20], [21]. The MECs after isolation were microscopically observed under the compound microscope to assess their purity. The purity of the isolated MECs were confirmed by reverse transcriptase (RT-PCR) using the MEC specific genes: cyotokeratin 8, α-lactalbumin, β-casein; myoepithelial cell: smooth muscle antigen; neutrophil: CD45; lymphocytes: CD19 and CD4; neutrophil chemotactic factor, IL-8, and housekeeping gene: β-actin as described by Janjanam et al., 2013 [13]. The final cell pellet was stored directly at −80°C for protein extraction or in 1 mL of Trizol reagent (Invitrogen) for RNA preparation.

### 4. Extraction of Total Proteins for 2D-DIGE

All chemicals, kits, instruments and data analysis software for the proteomics studies were from GE Healthcare, unless otherwise stated. Total proteins were extracted with the standard 2D-DIGE lysis buffer (7M Urea, 2M thiourea, 4% CHAPS and 30 mM Tris, pH: 8.5) containing protease inhibitor cocktail (Sigma, USA). Approximately 5–10×10^6^ cells were mixed with 200 µL of lysis buffer in micro centrifuge tube containing glass beads. The cells were mechanically disrupted using micro-pestle for 1 min and incubated on a rotator shaker for 30 min followed by centrifugation at 20,000 g for 30 min. The supernatant was transferred to a fresh tube and the protein sample was precipitated using 2D-Clean up kit according to the manufacturer's instructions. The final pellet was dissolved in 100 µL of same 2D-DIGE lysis buffer (labeling buffer). The pH of the protein samples were checked using pH indicator strips (Sigma, USA) and adjusted to nearly 8.5, and the protein concentration was determined by 2D-Quant kit.

### 5. CyDye Labeling

Proteins were labeled with minimal labeling method using DIGE-specific Cy2, Cy3 or Cy5 according to the manufacturer's instructions with minor modifications. Briefly after adjusting pH to 8.5, 50 µg of protein was mixed with 200 pmol of CyDyes and incubated on ice in the dark for at least 30 min. The reaction was stopped by addition of 1 µl of 10 mM lysine and incubated on ice for 10 min. All the samples were dye-swapped by labeling with both Cy3 and Cy5 to eliminate the dye-specific bias. For each DIGE gel 25 µg of each sample from two different samples were pooled and labeled with Cy2 for internal standard.

### 6. Two-dimensional Gel Electrophoresis

Pairs of Cy3- and Cy5-labeled protein samples were mixed together. Before rehydrating the IPG strips the unlabeled samples were spiked to 500 µg (250 µg from each sample per gel) to improve the mass spectrometry detection of low abundant proteins. The DIGE sample buffer (7M urea, 2M thiourea, 4% CHAPS, 2% DTT, and 1% IPG buffer) was added to bring the volume to 450 µL, and the samples were then applied to 24-cm Immobiline Drystrips for passive rehydration for overnight. IEF was carried out on an EttanIPGphor III at 20°C with maximum 75 µA/strip and the following setting: 150 V and 300 V each for 3 h, gradient increase to 1000 V in 6 h, 10,000 V in 1 h and final step at 10,000 V for 5 h reaching the desired total V-h. After IEF, IPG strips were equilibrated in equilibration buffer (6M urea, 30% (w/v) glycerol, 2% SDS, 50 mM Tris-HCl, pH 8.0) first with 1% DTT and then with 2.5% iodoacetamide each for 15 min. The strips were then transferred to 12.5% SDS-PAGE gels prepared by using EZ-Run 12.5% protein gel solution (Fisher Scientific, USA) for the second dimension electrophoresis using the EttanDalt Six gel system. Each set of plates were treated with Bind silane (spacer plates) and repel silane before casting the gels. SDS-PAGE was run at 1 Watt/gel for 1 h and then 3 Watts/gel for 15–20 h until the bromophenol blue dye front reached the gel end. A preparative gel was also run with pooled protein samples to pick protein spots of interest from analytical gels.

### 7. Image Acquisition

The gels were scanned using a Typhoon Trio+ variable mode imager using specific laser band-pass filters for each dye's excitation and emission wavelengths. The excitation and emission wavelengths were (480±35 nm) and (530±30 nm) for Cy2, (540±25 nm) and (590±35 nm) for Cy3, (620±30 nm) and (680±30 nm) for Cy5. The photomultiplier tube was set to 600 V, 575 V and 550 V for Cy2, Cy3 and Cy5 respectively for all the gels. The resolution was set to 100 µ with focal plane set to +3. All the gels were post stained with deep purple fluorescent dye which was performed essentially following the manufacturer's instructions, and subsequent scanning was performed using the same setting as that used for Cy3-labeled sample as well as reimaged post-excision to ensure accurate protein excision.

### 8. Image and Statistical Analysis

The DeCyder version 7.0 software tools were used for data normalization and analysis. The Differential In-gel Analysis (DIA) module was used to quantitatively compare the normalized volume ratio of each individual protein spot feature from a Cy3 or Cy5 labeled sample and directly quantified relative to the pooled internal standard sample of the Cy2 signal corresponding to the same spot feature. This was performed for all resolved images in a single gel where there was no gel-to-gel variation between the three co-resolved images, obviating the need to run technical replicates for each sample. The Biological Variation Analysis (BVA) was used collectively from the DIA datasets for each individual gel where Cy2 standard were used to normalize and compare Cy3:Cy2 and Cy5:Cy2 abundance ratios across each six-gel set. Statistical confidence was associated with charge-altering post-translational modification or change in abundance using Student's t test and the variation of expression within a group to the level of change between groups was analyzed by analysis of variance (ANOVA). The statistical significant changes within 95% confidence were considered for further analysis. Unsupervised principal component analysis (PCA) and Hierarchical Clustering (HC) analyses were performed using the Extended Data Analysis (EDA) module of DeCyder 7.0. These multivariate analyses clustered the individual Cy3 and Cy5 labeled samples based on the collective comparison of expression patterns from the proteins identified in our study. The protein interaction network and pathways associated with identified proteins were generated through the use of IPA (Ingenuity® Systems, www.ingenuity.com).

### 9. In-gel Digestion, Mass Spectrometry, and Database Interrogation

Proteins of interest were robotically excised using Ettan Spot picker and digested into peptides by in-gel digestion with modified trypsin (Trypsin Gold, Promega). The mass spectrometry analysis and database interrogation was followed as described previously by Bringans et al., 2008 [22]. MALDI TOF/TOF analysis of protein spots was performed on a 5800 MALDI TOF/TOF analyzer (AB Sciex). Excised deep purple fluorescent stained gel spots were de-stained by three 45-min washes with 25 mM ammonium bicarbonate in 50∶50 ACN∶water. De-stained and washed gel pieces were vacuum-dried and stored at −20°C until tryptic digestion. Ten µL trypsin digest solution (12.5 ng/µL trypsin, 25 mM ammonium bicarbonate) was added to each gel piece and incubated overnight at 37°C. The digested peptides were extracted by two 20-min incubations with 10–20 µL ACN containing 1% TFA, depending on the size of the gel piece. Exceptionally, three extractions were applied for large-size gel pieces. The pooled extracts were dried by rotary evaporation and stored at −20°C pending further analysis by MS. The dry samples were reconstituted in 1 mL of 0.1% formic acid and then further diluted 1∶100 in 50∶50 acetonitrile/water. The resulting solution was spotted 1∶1 with matrix solution (α-cyanohydroxycinnamic acid; 5 mg/mL) in duplicate on a 384-well Opti-TOF stainless steel plate (AB Sciex). The spotted samples were analysed using a first run of standard TOF MS. The system was set to perform MS/MS analysis focused on the 20 most intensive peaks of the first MS run (excluding peaks known to be trypsin). The laser was set to fire 400 times per spot in MS mode and 2000 times per spot in MS/MS mode with laser intensity at 3300 J (MS) and 4200 J (MS/MS). A mass range of 800–4000 amu with a focus mass of 2100 amu was used.

Low abundant proteins which couldn't be identified by MALDI-TOF-TOF were subjected to ESI-LC-MS/MS. Electrospray mass spectrometry was performed on a 4000 Q-TRAP mass spectrometer (Applied Biosystems) with sample introduction from an Ultimate 3000 nanoflow LC system (Dionex, Bannockburn, IL, USA). Protein digests were eluted from a Pepmap C18 column (Dionex) with an increasing gradient of acetonitrile with mobile phase containing 0.1% formic acid at 300 nL/min. The mass spectrometer was set to perform an ‘information- dependent acquisition’ (IDA) method to acquire fragmentation data on the three most intense ions that met the following criteria: mass range 400–2800, charge 1–3, intensity greater than 5e4 counts, exclusion of former target ions for 300 s.

### 10. Real Time Quantitative RT-PCR

Total RNA from mammary epithelial cells of different experimental conditions was isolated using Trizol reagent (Invitrogen) and treated with DNAse using DNA-free kit (Ambion, Life Technologies) to remove the DNA contamination. Quantitative PCR of mRNA from purified MECs was carried out using Light Cycler 480 SYBR Green I Master technology (Roche Diagnostics) as reported previously [13]. The relative expression was expressed in terms of fold change after calculating the expression index (ΔΔCp) and the differences were considered to be statistically significant at p<0.05. All the primers' sequences used for real-time PCR analysis were listed in supporting information table S1.

### 11. Western blot Analysis

Western blot analysis was performed as described previously [13], [23]. The protein concentration was determined by using 2-D Quant kit (GE Healthcare, USA). The proteins were pooled from each group and 20 µg was loaded per well. The blots were incubated in blocking solution (NAP blocker, G-biosciences) at 4°C overnight. The blots were incubated with primary antibodies at 1∶1000 dilution for Annexin A1 and ARP3 (Annexin- Sigma, SAB2500072-100UG & ARP3- Thermo Scientific, Pierce, PA5-30354), 1∶500 dilution for PGAM1 and Lamin B1 (PGAM1- MyBioSource.com, MBS421435 & Lamin B1- Thermo Scientific, Pierce, MA1-06103) and 1∶2000 dilution for Vimentin (Santa Cruz Biotechnology, Inc) followed by secondary antibodies at 1∶2000 dilution (Merck). The blots were developed using DAB system (Merck).

## Results and Discussion

### 1. Protein expression profile at different stages of lactation & milk yielding capacity

**Figure 1:**
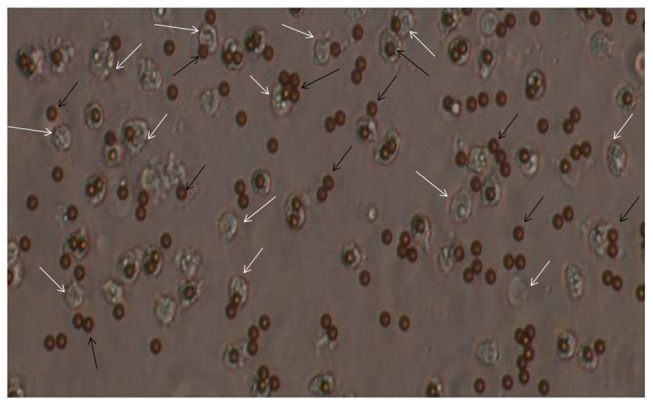
MECs isolation from milk using immunomagnetic beads. The purified MECs which were separated from other somatic cells using immunomagnetic beads coated with anti-cytokeratin antibodies specific to mammary epithelial cells and observed under a microscope at 200×. White arrows indicate MECs and black arrows indicate immunomagnetic beads.

**Figure 2:**
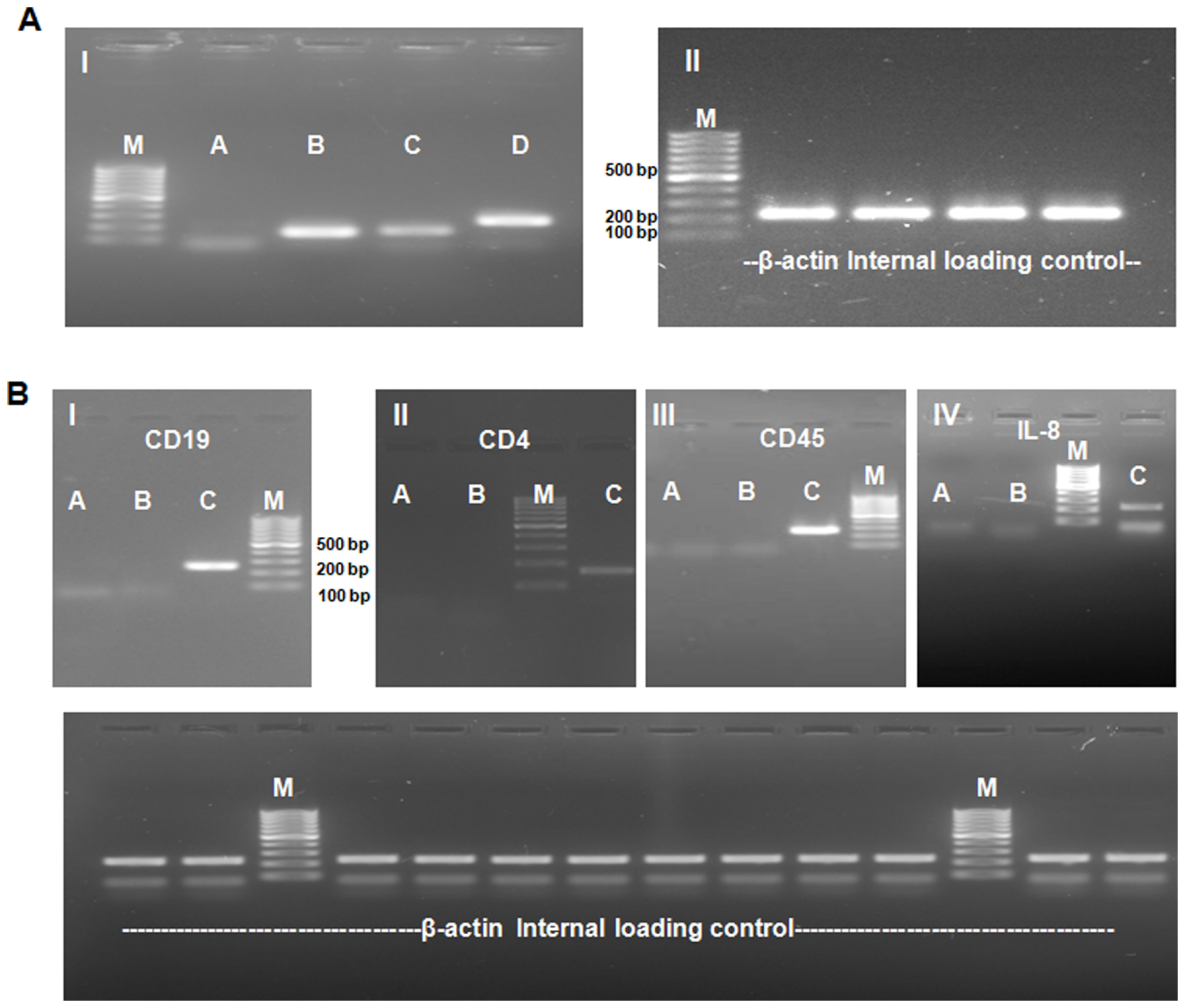
RT-PCR analysis of Bovine Mammary Epithelial cells. MECs specific gene's primers (I); M: 100 bp ladder; A: SMA; B: Cytokeratin 8; C: β-casien and D: α-lactalbumin; (II): Loading control represents the house keeping gene β-actin (A). Other cell types using for CD19 (I), CD4 (II), CD45 (III) and IL-8 (IV); M: 100 bp ladder; A: Skin fibroblast (Negative control); B: Mammary Epithelial cells and C: Somatic cells (Positive control); Loading control represents the house keeping gene β-actin (B).

**Figure 3:**
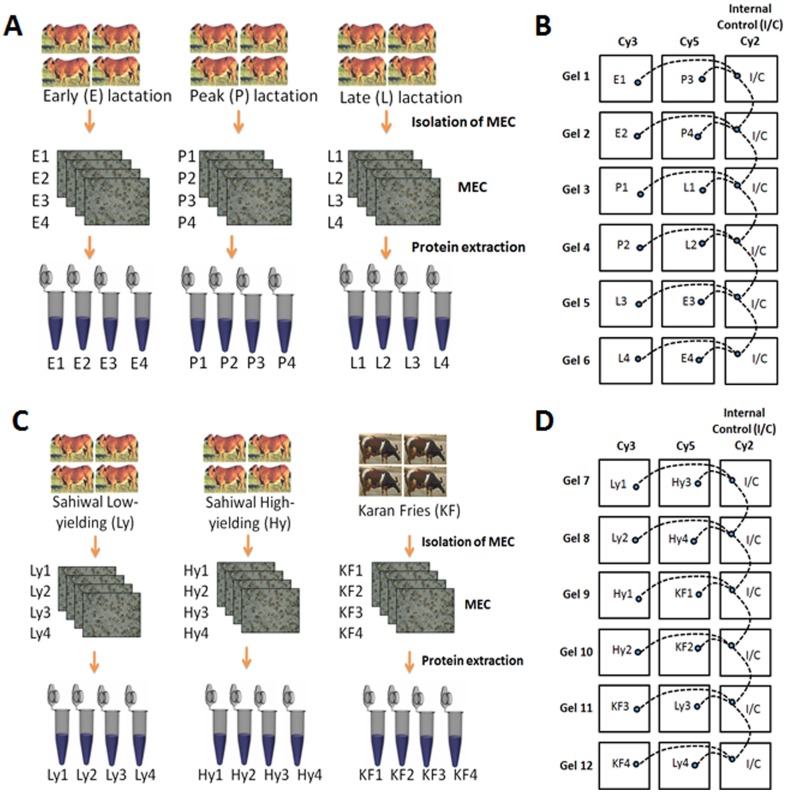
Schematic representation of 2D-DIGE experimental design. Comparative proteome analysis of bovine mammary epithelial cells at Early, Peak and Late stages of lactation (A) and CyDye labeling and dye swapping for early, peak and late stage samples (B). Schematic representation of 2D-DIGE experimental plan for comparative proteome analysis of bovine MEC isolated from high and low milk yielding animals (C) and CyDye labeling for corresponding samples (D).

**Figure 4:**
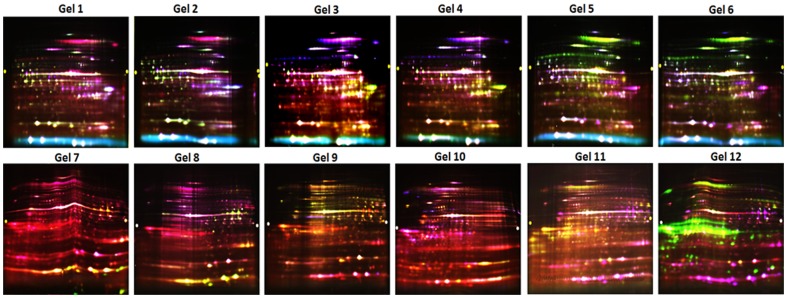
2D-DIGE gel images. All 12 gels of both experiments (gels 1–6 represents early vs peak vs late lactation; gels 7–12 represents low yielding Sahiwal cows vs high yielding Sahiwal cows vs high yielding Karan Fries (KF) cross bred cows) represented above. The gels were scanned using all the three lasers corresponding to Cy2, Cy3 and Cy5 wave lengths. The images were taken at 200 µ resolution. The green color spots are down regulating and red color spots are up regulating proteins.

MECs after isolation were observed in a compound microscope. The isolated MECs looked quite homogeneous with most of the cells attaching to dynabeads (Figure 1). We observed amplification of MEC specific genes such as cytokeratin 8, α-lactalbumin and β-casein while no amplification took place in genes belonging to other cell types such as lymphocyte (CD19 & CD4) and leucocyte (CD45 & IL8) (Figure 2). The MECs from each group of animals were isolated and the proteins were extracted as shown in Figure 3. To assess the changes in protein profile in these samples, DIGE/MS analysis was performed using a pooled sample internal standard present in every gel (Figure 3B & 3D). In each experiment, every DIGE gel contained an equal aliquot of all the 12 samples labeled as Cy2 (internal standard). The proteins labeled with Cy3 and Cy5 were compared in each gel and normalized with Cy2 labeled samples. The experimental design and the DIGE analysis were followed as described by Friedman et al., 2007 [24]. From the 6 gels of each experiment a total of 2523 proteins from different stages of lactation (Gel 1–6, Figure 4) and 2672 proteins from high and low-yielding samples (Gel 7–12, Figure 4) were resolved and matched across all 6 gels of each experiment. These resolved protein features included many isoforms that resulted from charged post-translational modifications and/or processing. The differential in gel analysis (DIA) provided room for direct measurements of expression levels for each resolved protein within a gel. These spot maps were obtained from Cy3 and Cy5 channels normalized to Cy2 channel independently that eliminates technical variation due to co-migration of all tripartite labeled proteins. This methodology enabled normalization of the Cy3/Cy2 and Cy5/Cy2 intra gel ratios between gels by virtue of the signal from the Cy2 internal standard, which was present in each gel. This analysis was performed independently for the 2523 and 2672 resolved features of each experimental set. Biological variation analysis (BVA) was carried out to compare variation within the group where all the proteins were statistically significant. DIGE analysis of MEC at different stages of lactation flagged 93 protein forms and differential expression among high and low-milk yielding samples resulted in 72 protein forms with statistical significance. The changes in abundance or charge-altering post-translational modification that were greater than 1.5 fold difference includes 41 unique proteins with regard to different stages of lactation (Table 1) and 22 unique protein with regard to high and low-milk yielding groups (Table 2). Many of these unique proteins migrate as additional charge-related isoforms with indistinguishable molecular masses with charge-altering post-translational modifications. The expression patterns of these isoforms were overall similar to those listed in Table 1, 2 and in most cases these were omitted from the table for brevity. The information details of the protein identification and peptide details were listed in the supporting information tables S2 and S3. Among the 93 differentially expressed protein spots at different lactation stages, we could identify only 83 protein spots (Table S2). Whereas, among the 72 protein spots that were differentially regulated in high and low yielding samples, we could identify only 35 protein spots (Table S3). This is due to either we couldn't obtain significant spectral data or the PMF data didn't match with database for rest of these proteins. Proteins with expression changes within the 95^th^ percentile were only considered for statistical significance. The protein spots were picked from both analytical gels and also preparative gel run with pooled protein samples that were matched with the master gel in BVA analysis. The protein spots of interest were assigned and exported to Ettan spot picker. The present analysis used medium range pH 4–7 gradients for the first dimension isoelectric focusing because this condition offers increased resolution and sensitivity compared with broader range gradients. [25].

**Table 1:** Differentially expressed proteins at different stages of lactation were identified by MALDI-TOF/TOF or LC-MS/MS.

S. No	Spot ID	Protein Name	Early vs Peak (P/E)		Peak vs Late (L/P)		Early vs Late (L/E)	
			Av. Ratio	T-test	Av. Ratio	T-test	Av. Ratio	T-test
1	773	Aldehyde dehydrogenase, mitochondrial	−1.53	3.80E-05	1.61	0.00011	1.06	0.061
2	883	Alpha-enolase	−2.14	4.00E-05	1.01	0.58	−2.13	1.60E-04
3	1376	alpha-S1-caseinor	1.54	0.00067	−4.57	8.60E-05	−2.96	3.00E-04
4	1174	Alpha-S2-casein	−1.19	0.063	−3.85	0.0001	−4.58	1.00E-04
5	1281	Annexin 5	2.47	8.70E-05	−1.84	0.00028	1.34	0.0032
6	1133	annexin A1	−3.71	4.00E-05	1.46	0.011	−2.54	0.0001
7	1245	Annexin A3	−2.2	5.10E-05	1.71	0.00077	−1.29	0.003
8	844	ARP3 actin-related protein 3 homolog	−2.8	0.00058	2.77	0.001	−1.01	0.31
9	1214	Beta-actin (Fragment)	1.48	0.00075	1.94	0.00015	2.86	5.70E-06
10	1410	Beta-casein (Fragment)	1.24	0.02	−4.63	7.70E-05	−3.73	2.20E-05
11	2160	Beta-lactoglobulin	1.26	0.027	1.15	0.02	1.45	0.0073
12	660	CALR protein	1.65	0.00072	1.03	0.41	1.7	1.60E-04
13	1932	Cathelicidin-1 Precursor	1.77	0.00065	−1.47	0.003	1.2	0.013
14	1952	Cathelicidin-6	−1.33	0.051	−2.85	0.011	−3.79	4.30E-04
15	1330	cytoskeletal beta actin	3.19	4.10E-05	−1.88	0.00027	1.7	0.00049
16	1202	F-actin-capping protein subunit alpha-1 (CapZ alpha-1)	−1.3	0.0046	1.6	0.0012	1.23	0.026
17	932	Gelsolin	3.17	0.00023	−1.12	0.016	2.82	3.10E-04
18	807	Guanine Deaminase	−1.8	0.00099	1.71	0.00081	−1.05	0.12
19	1551	Haptoglobin (Fragment)	−1.57	0.00047	−1.59	0.01	−2.5	0.00019
20	1420	hypothetical protein	8.18	0.00047	−21.4	4.80E-05	−2.62	0.02
21	614	Keratin, type I cytoskeletal 25	−1.01	0.15	1.62	8.00E-05	1.61	5.70E-06
22	423	lamin-B1	1.38	0.0021	1.35	0.0069	1.86	0.00033
23	764	MFGE8 protein	−1.35	0.007	1.68	0.0054	1.24	0.008
24	833	PDIA6 protein	1.26	0.0019	1.27	0.005	1.6	7.00E-05
25	1745	Peroxiredoxin-5, mitochondrial	−1.33	0.0021	−1.42	0.00059	−1.89	8.00E-05
26	1434	Phosphoglycerate mutase	−1.58	0.0001	1.4	4.00E-05	−1.13	1.10E-02
27	1339	PREDICTED: GDP-L-fucose synthase-like	1.02	0.15	−1.95	0.00037	−1.92	0.00084
28	1071	PREDICTED: heat shock 70kDa protein 8	1.34	0.0094	−1.83	0.0002	−1.37	0.008
29	1049	PREDICTED: serpin peptidase inhibitor, clade B like	16.09	2.70E-05	−14.6	7.60E-06	1.1	1.80E-01
30	1556	proteasome subunit beta type-3	−1.48	6.90E-05	−1.75	1.90E-05	−2.59	2.00E-06
31	643	Protein disulfide isomerase-associated 3	−1.37	0.022	3.45	0.00028	2.51	0.00078
32	642	protein disulfide-isomerase A3 precursor	−1.66	0.0025	2.97	6.20E-05	1.8	0.00078
33	622	Protein S100-A8	−1.31	0.028	1.8	0.0022	1.37	0.034
34	1462	Putative uncharacterized protein	−1.27	0.0016	−1.07	0.3	−1.36	0.0049
35	1424	Rho GDP-dissociation inhibitor 2	−1.22	0.026	−2.1	0.00074	−2.56	2.10E-05
36	1179	Serpin B1	−3.98	2.80E-05	3.49	3.90E-05	−1.14	0.022
37	1009	SERPINB4 protein	−3.13	8.70E-05	1.7	0.0016	−1.84	0.00036
38	2157	Thiopurine S-methyltransferase	1.62	0.0016	2.31	5.10E-05	3.73	2.80E-05
39	743	Tubulin alpha chain	−1.46	0.00019	−1.64	0.00022	−2.4	2.30E-05
40	1076	Uncharacterized protein	1.22	0.029	−1.64	0.016	−1.34	0.023
41	1029	uncharacterized protein (Actin-related protein 2)	−2.09	2.70E-04	1.45	0.012	−1.44	0.0025

**Table 2:** Differentially expressed proteins in MEC of high and low yielding animals were identified by MALDI-TOF/TOF or LC-MS/MS.

			Hy vs Ly (Hy/Ly)		Ly vs KF (Ly/KF)		Hy vs KF (Hy/KF)		
S. No.	Spot ID	Protein Name	Av. Ratio	T-test	Av. Ratio	T-test	Av. Ratio	T-test	1- way ANOVA
1	589	Keratin 5	−2.2	0.019	2.94	0.1	1.34	0.14	0.002
2	594	Moesin	−2.58	0.0077	3.65	0.066	1.41	0.13	0.002
3	726	PDIA3 protein	1.65	0.0091	−1.4	0.4	1.18	0.31	0.05
4	739	Keratin 10	2.09	0.0091	−2.28	0.25	−1.09	0.44	0.042
5	770	CALR protein	1.31	0.013	1.55	0.3	2.02	0.13	0.024
6	800	Aldehyde dehydrogenase, mitochondrial	1.82	0.031	−1.76	0.36	1.03	0.41	0.043
7	818	Milk fat globule-EGF factor 8	3.68	0.029	−3.94	0.066	−1.47	0.22	0.011
8	891	Alpha-enolase	−1.98	0.079	4.7	0.14	2.38	0.21	0.026
9	903	Adenosylhomocysteinase	−1.53	0.21	3.11	0.12	2.03	0.22	0.036
10	948	Serpin peptidase inhibitor, clade B like	−8.57	0.0091	8.72	0.096	1.02	0.44	0.008
11	952	β-actin	−3.82	0.0091	3.79	0.1	−1.01	0.45	0.007
12	967	Alpha 2 actin	−1.65	0.022	1.75	0.25	1.06	0.4	0.046
13	1037	Annexin A1	−2.17	0.023	3.31	0.066	1.53	0.13	0.008
14	1382	KRT18 protein	−1.55	0.048	1.92	0.17	1.24	0.29	0.034
15	1460	Protein S100-A9	−2.55	0.047	1.54	0.43	−1.65	0.24	0.054
16	1722	KRT15 protein	−2.5	0.035	1.65	0.39	−1.52	0.31	0.058
17	1801	RAGE-binding protein	−3.05	0.035	1.8	0.27	−1.69	0.24	0.031
18	1809	Protein S100-A8	−3.51	0.01	1.74	0.37	−2.02	0.23	0.008
19	2014	Transaldolase	−1.45	0.13	1.97	0.21	1.36	0.2	0.04
20	2018	Vimentin	−2.52	0.028	6.84	0.14	2.72	0.19	0.038
21	2021	Protein S100-A12	−4.02	0.019	2.3	0.25	−1.75	0.27	0.026
22	2022	Alpha-S1-casein	1.94	0.023	−5.01	0.31	−2.58	0.3	0.092

### 2. Principal Component Analysis (PCA) and Hierarchical Clustering (HC) analysis

**Figure 5:**
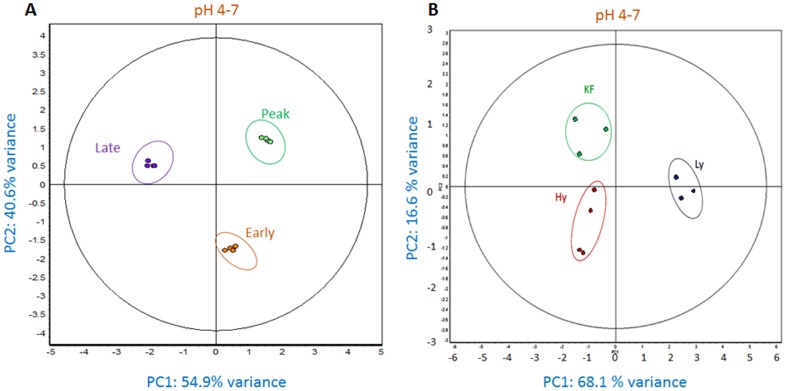
Principal Component Analysis (PCA) of spot maps. The different stages of lactation (A) and high and low-yielding samples (B) to represent high reproducibility among biological samples within each group.

**Figure 6:**
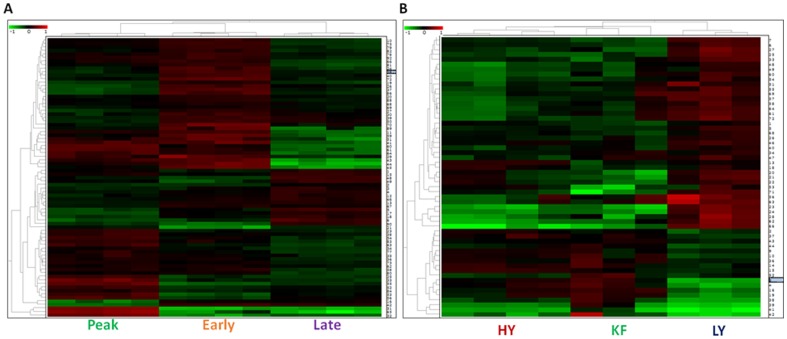
Unsupervised Hierarchical Clustering (HC). The expression maps and spot maps with relative expression values for each are displayed as heat map using a relative scale from −1 (green) to +1 (red). A. HC of total differentially regulated proteins identified at different stages of lactation and B. HC of total differentially regulated proteins identified from high and low milk yielding samples.

Unsupervised PCA and HC were performed to further validate the experimental samples for relevance of differentially expressed genes. These multivariate statistical tests and network mapping of identified proteins establish biological significance of the resulting protein changes. PCA reduces the dimensionality of a multidimensional analysis to display the two principal components that distinguish between the two large sources of variation within the dataset. PCA indicated distinct expression patterns from the three groups of each experiment and demonstrated high reproducibility between the replicate samples (Figure 5). Each data point in the PCA plots describes the collective expression profiles for the subset of proteins identified. The differentially expressed features identified at different stages of lactation, the first principal component distinguished 54.9% of the variance with 40.6% additional variation distinguished by the second principal component. Whereas the differentially expressed proteins of high and low yielding samples represent 68.1% and 16.6% of PC1 and PC2 respectively. In addition, the PCAs demonstrate that the greatest amounts of variation in the experiments are what distinguish the late stage from other two stages and low-yielder samples from both high-yielding and KF samples. These grouping assignments were reiterated in an unsupervised HC analysis of the protein expression patterns within each sample from the experiments (Figure 6). HC performs a similar clustering of the samples based on similarities of expression patterns in the selected proteins, which are visually presented as horizontal lines in an expression matrix “heat map” using a standardized log abundance scale ranging from −1.0 (green) to +1.0 (red). HC expression matrices were calculated using Euclidean correlation and average linkage. Each column in the HC expression matrix is effectively the same as each data point in the PCA plots. The PCA and HC results validate the biological significance of the protein expression changes identified in both the experimental groups. We would not expect these individual samples to cluster in this way if the changes arose stochastically. Some of the differentially expressed spots were graphically represented in supporting figure S1.

### 3. Functional groups of regulated proteins

On the basis of the available literatures reported by different authors, the possible functional significance of few differentially expressed proteins have been outlined. Ninety three proteins were selected for identification as they were observed at significantly higher or lower levels between different stages of lactation i.e. early, peak and late. Out of 93 selected proteins, 10 proteins did not match with the database and we were unable to connect their protein identity. The remaining 83 identified proteins include isoforms and post-translational modification which resulted in 41 unique proteins. These identified proteins followed varying expression pattern between different stages of lactation (Table 1). During late lactation decline in milk production takes place due to decrease in MEC numbers and also secretory activity of MEC [26]. Up-regulation of these proteins during late lactation may be playing significant role in decline of milk yield after peak lactation by getting involved in apoptosis of MEC, blocking milk secretion pathways and by negatively regulating metabolic pathways which are essential for milk production and maintenance of lactation persistency. The most strongly regulated proteins which we observed were cytoskeletal components, regulators of protein folding and stability, calcium-binding proteins, and regulators of cellular metabolism. There was preferential expression of regulation of protein stability, such as serpins and serpin-like proteins, disulfide isomerase and subunits of the proteasome in peak and late stages of lactation. Up-regulation of Serpin clade B-like protein during peak stage of lactation and up-regulation of serpin B1 and B4 during early stage of lactation indicates that suppression of proteolysis may have been necessary to allow the emergence of nutritionally functional proteins, particularly those with little stabilizing secondary and tertiary structure [27], [28]. In case MEC isolated from high and low-milk yielding animals, 35 proteins were identified which include isoforms and post-translation modifications that resulted in 22 unique proteins (Table 2). Among these, the most strongly regulated proteins are cytoskeletal components, calcium-binding proteins, regulators of cellular metabolism and regulators of protein stability.

Cytoskeletal proteins are up-regulated or repressed in MEC at different stages of lactation. The data shows heat shock 70 kDa protein 8 up-regulating in peak stage of lactation. HSP family of chaperones may play a role in the formation and function of the cytoskeleton [29]. Interestingly the data shows that Cytoskeletal beta actin up-regulated 3.2 folds in the peak stage of lactation as compared to other stages of lactation. Rearrangement of the actin cytoskeleton is very important for the complex process of cell polarization [30], [31]. Polarity of the MEC during lactation plays crucial role for milk production and secretory activities. Interestingly we also observed that equal level of gelsolin was up-regulated (3.17 fold) which supports the above observation. Gelsolin is the founding member of a family of actin-binding proteins involved in controlling the organization of actin cytoskeleton in cells [32]. Gelsolin severs actin filament in the presence of micromolar amount of calcium, thereby disassembling the actin network. Although embryonic development and longevity were normal in case of mice lacking gelsolin, migration of neutrophils and dermal fibroblasts was decreased [33] indicating that gelsolin is required for rapid motile response in some cell types, such as those involved in homeostasis, inflammation, and wound healing. Lack of gelsolin resulted in defects in mammary gland development [34]. The requirement of gelsolin for ductal development of the mammary epithelium is not surprising. Gelsolin mediates epidermal growth factor (EGF) effects on cell motility [35] and EGF controls ductal outgrowth, elongation and branching [36], [37]. The present study shows enhanced expression of gelsolin during peak stage of lactation. This supports that the polarity of MEC and cell shape were maintained which is important for the milk secretion and export.

Expression of proteasome subunit beta type-3 was up-regulated during early stage and the expression levels were reduced in peak stage and least in late stage of lactation. The proteasome is the major proteolytic complex of eukaryotic cells, primarily essential for degradation of polyubiquitinated proteins. Our data show that the expression of proteasome subunit beta type 3 was gradually decreasing from early stage of lactation to the late stage of lactation. The proteasome has been implicated in the proteolysis of transcription factors, cell cycle regulatory proteins, oncogenes, tumor suppressors, and proteins involved in cellular differentiation [38]–[41]. The rapid degradation of certain regulatory proteins is important in the stringent control of their signaling activity [38], [39]. Our results suggest that activity of proteasome may be modulated by differential expression of specific subunit components during lactation process.

A family of calcium-binding proteins (Annexins) was highly regulated at different stages of lactation. These proteins are involved in many cellular processes, and binding to calcium regulates their activity. Annexins are phospholipid-binding proteins that may cross-link plasma membrane phospholipids, actin, and the cytoskeleton [42] to regulate proliferation, [43] exocytosis [44], membrane fusion [45] and also present in milk exosomes [46]. Annexin A1 expression correlates with cell proliferation and it's over expression blocks intracellular calcium release and inhibits cell adhesion and differentiation [47]. Annexin A5 expression was up-regulated in peak and late stage of lactation which is opposite to that of annexin A1 profile. In case of annexin A3, it was down regulated in peak and up-regulated in both early and late stages. The results suggest that different forms of annexins are regulated differently during different stages of lactation for maintaining cell number, exocytosis and milk secretion.

In the present study an up-regulation of Serpin clade B-like protein up to 8.5 fold was observed in low yielding cows as compared to both Sahiwal and Karan-fries high yielding cows. Serpin clade B proteins are mostly intracellular and play important role in protecting the cells from proteases. While most serpins control proteolytic cascades, certain serpins do not inhibit enzymes, but instead perform diverse functions such as storage (ovalbumin, in egg white), hormone carriage proteins (thyroxine-binding globulin, cortisol-binding globulin) and tumor suppressor genes (maspin). The term *serpin* is used to describe these latter members as well, despite their non-inhibitory function [48], [49]. As serpins control processes such as coagulation and inflammation, these proteins are the target of medical research. However, serpins are also of particular interest to the structural biology and protein folding communities, because they undergo a unique and dramatic change in shape (or conformational change) when they inhibit target proteases [49]. Our results suggest that up-regulation of serpin clade B-like protein might have non-inhibitory function and probably function as hormone carriage protein like thyroxine or cortisol-binding, which may lead to shifting of energy balance and thereby increasing in metabolic rate. Cortisol may also cause increased glucose levels and promote break down of proteins. These effects might be lowering the milk production in low producers which need to be validated by functional studies of these serpin clade B proteins. Serpin A3-1 and few other proteins were reported as up-regulated in mastitis condition [50] however we didn't find any of these protein in our present study.

Milk fat globule-EGF factor 8 (MFGE8 also known as lactadherin) is significantly up-regulated (3.5 fold) in both Sahiwal and Karan fries high yielders. MFGE8 has a domain similar to epidermal growth factor and MFGE8 promotes RGD-dependent cell adhesion via integrins [51]. Integrins intern helps in regulating mammary gland proliferation and maintains the integrity of mammary alveoli [52]. The results suggest that up-regulation of MFGE8 is important for alveolar integrity and high milk production in cows. In the present study four proteins belong to S100 protein family, includes S100-A8, S100-A9, S100-A12 and RAGE-binding protein. Interestingly all these proteins were up-regulating in low yielding sahiwal cows as compared to both high yielding Sahiwal as well as Karn-fries cows. The name is derived from the fact that the protein is 100% soluble in ammonium sulfate at neutral pH. S100 proteins have been implicated in a variety of intracellular and extracellular functions [53]. S100 proteins are involved in regulation of protein phosphorylation, transcription factors, Ca++ homeostasis, the dynamics of cytoskeleton constituents, enzyme activities, cell growth and differentiation, and inflammatory response. S100-A8 and S100-A9 may function in the inhibition of casein kinase which plays significant role in TGF signalling pathway. It inhibits apoptosis caused by TGF by blocking TGF pathway in mammary gland [54]. Up-regulation of S100 proteins in low producing animals results in inhibition of casein kinase which helps in cell survival and continuance of milk synthesis and secretion. The present finding indicates that up-regulation of S100 proteins in low producing animals ultimately causing apoptosis decreases the ability to maintain the optimum MEC numbers resulting in decreased milk production. S100 proteins have also been reported to bind to intracellular calcium in MEC. Calcium is essential for milk secretion [55]. Binding of S100 proteins to calcium inside MEC will make calcium unavailable for secretion activity resulting in decreased milk yield. Therefore S100 proteins may be playing a major role in decreasing milk production in low producing animals.

### 4. Bioinformatics analysis of differentially expressed proteins

**Figure 7:**
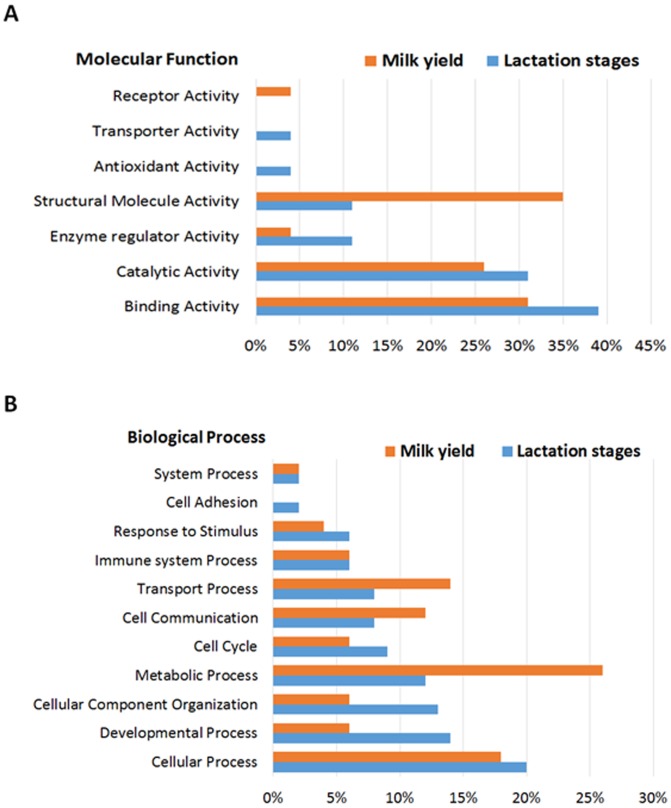
Categorization of identified proteins based on molecular function and biological process. Differentially expressed proteins identified were categorized based on Molecular function (A) and Biological process (B) using panther classification system [67].

The differentially expressed proteins of bovine MEC from the above experiments were distributed into categories with regard to their molecular function and biological processes using PANTHER classification system. Categorization based on molecular function showed that more than half of identified bovine MEC proteins accounted for binding and catalytic activity which are essential for cell to cell interaction and metabolic activity. Cell-matrix interaction plays role in growth, development and remodeling of mammary gland throughout the stages of lactation. Rest other proteins were found to be involved in structural molecule activity, enzyme regulator activity, anti-oxidant activity, transporter activity and receptor activity (Figure 7A). The proteins classified based on biological processes display one fourth of identified proteins in metabolic process and others were involved in cell communication, cellular process, transport, cellular component organization, cell cycle, immune system process, response to stimulus, cell adhesion and system process (Figure 7B). The present findings suggests that proteins involved in metabolic and signaling pathways were differentially expressing in MEC during different stages of lactation and varying milk yielding capability of MEC.

**Figure 8:**
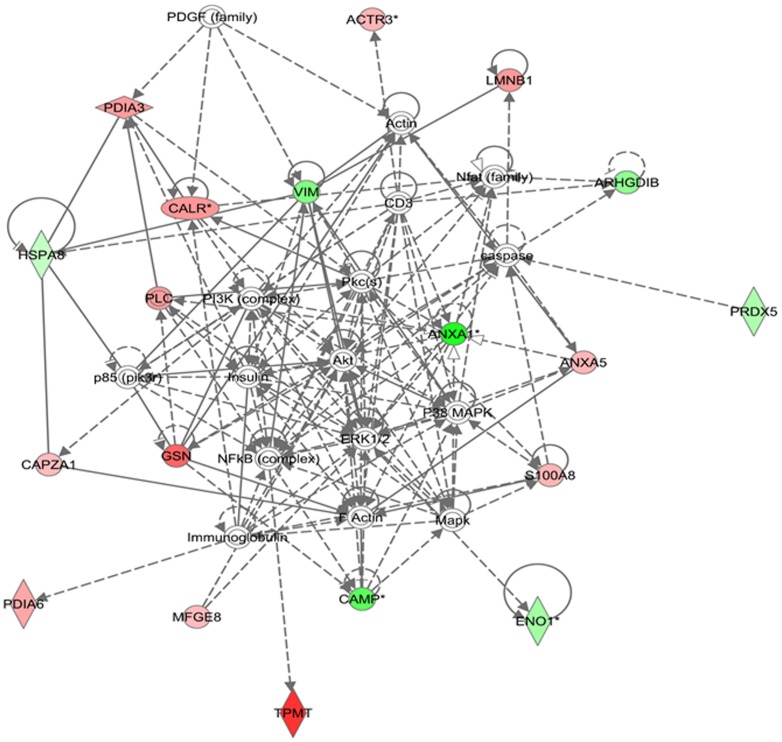
Network analysis of differentially expressed proteins. The differentially expressed proteins were mapped onto existing mammalian pathways and networks of protein-protein interactions and other biochemical pathways reported previously in the literature. In this network 1, Red color features are the up-regulated proteins during peak stage and green color features are down-regulated during early stage of lactation. The full names of these proteins are shown in supporting information table S4.

**Figure 9:**
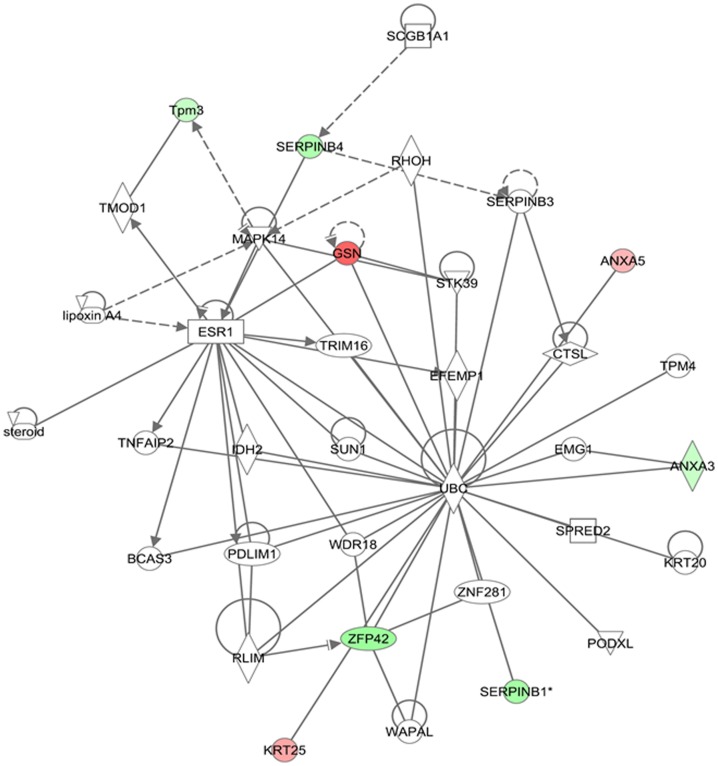
Pathway analysis of differentially expressed proteins at early and late stages of lactation. The differentially expressed proteins during early and late stages were mapped onto existing mammalian pathways and networks and in this network 2, the red color proteins represent up-regulated in late and down-regulated in early stages of lactation.

**Figure 10:**
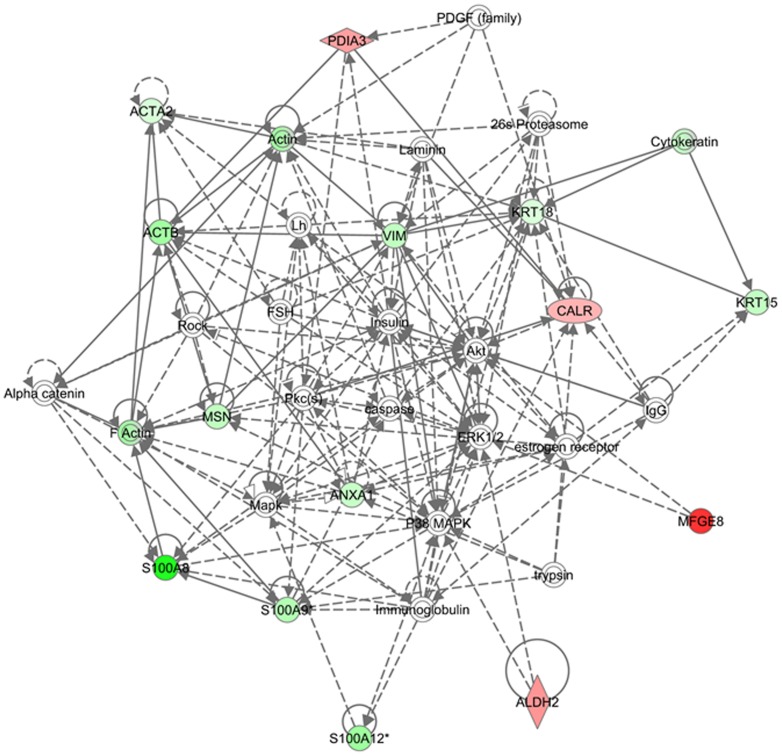
Pathway analysis of differentially expressed proteins in high and low milk yielding cows. The differentially regulated proteins of high and low milk yielding samples were mapped onto existing mammalian pathways and networks. In this network 3, the red color proteins are up-regulated in high-yielding (Hy) and down-regulating in low-yielding (Ly) samples. The full names of these proteins are shown in supporting information table S5.

The network analysis of differentially expressed proteins during lactation stages resulted in an interaction network and pathways. The proteins mapped in these networks were mostly involved in molecular transport, cell morphology, cell-to-cell signaling and interaction (Figure 8). In the network and pathway analysis, Akt, PI3K, p38/MAPK and nuclear factor kappa B (NF-κB) were identified as major hub connecting most of these proteins either directly or indirectly. It was reported that PI3K/Akt pathway is important during lactation for synthesis of milk components, especially lipids and lactose [56], [57]. p38 MAPK helps in increasing protein synthesis by stabilizing the mRNAs mediated via AU-rich element-binding protein (ARE-BP) phosphorylation [58]. In a recent study, it was reported that p38 MAPK expression significantly increases through the initial four months of lactation [59]. These results indicate that proteins up-regulated in the peak stages shown in red interact with the PI3K/Akt pathways either directly or indirectly thus helping in increasing synthesis of lactose and lipids or interact with p38 MAPK pathways to enhance syntheses of milk proteins. NF-κB activity important regulatory role at different stages of mammary development. It was reported that NF-κB activity increases during pregnancy and involution and decreases during lactation [60], [61]. Connelly et al., reported that activation of NF-κB signaling pathway during lactation results in milk clearance and apoptosis [62]. Interestingly, most of these proteins were up-regulated during late stage of lactation. This indicates that during late stage of lactation the mammary epithelial cells undergo apoptosis and cell death which results in decline in milk production. In another network the major proteins interact directly or indirectly to ubiquitin C and MPK14 (p38-α) (Figure 9). The ubiquitin C is a polyubiquitin precursor which has various effects within a cell and the process of ubiquitination is associated with protein degradation, cell cycle regulation, endocytosis and regulation of cell signaling pathways. The ubiquitination of NF-κB inhibitors results in activation of NF-κB signaling pathway [63]. The Jak and MPK14 are associated with signaling pathways activated during cell stress [64], [65]. This network analyses indicates that the up-regulation of GSN, ANXA5 and KRT25 proteins during late stage of lactation may trigger the activation of MAPK14 associated during cell stress and apoptosis. This indicates that the late stage could be the more stressful stage during lactation and results in declining in milk production. Activation of these pathways may have a major impact on lactation persistency in bovine and human alike. The network analysis of differentially expressed proteins in MEC isolated from high and low milk yielding samples are represented in Figure 10. The up-regulated protein in high-milk yielding samples, PDIA3, CALR, MFGE8 and ALDH2 were connected through Akt and p38 MAPK pathways thus helping in milk production in high producers. Interestingly, most of the interactions in these networks were mediated through insulin. Thus insulin hormone signaling plays important role in determining the milk yielding capacity of MEC. This finding is also supported from a recent study on transcriptome of milk fat layer at different stages of lactation which reported that insulin is important for milk synthesis [66].

### 5. Validation of proteomics data with Western blot and Real-time PCR

**Figure 11:**
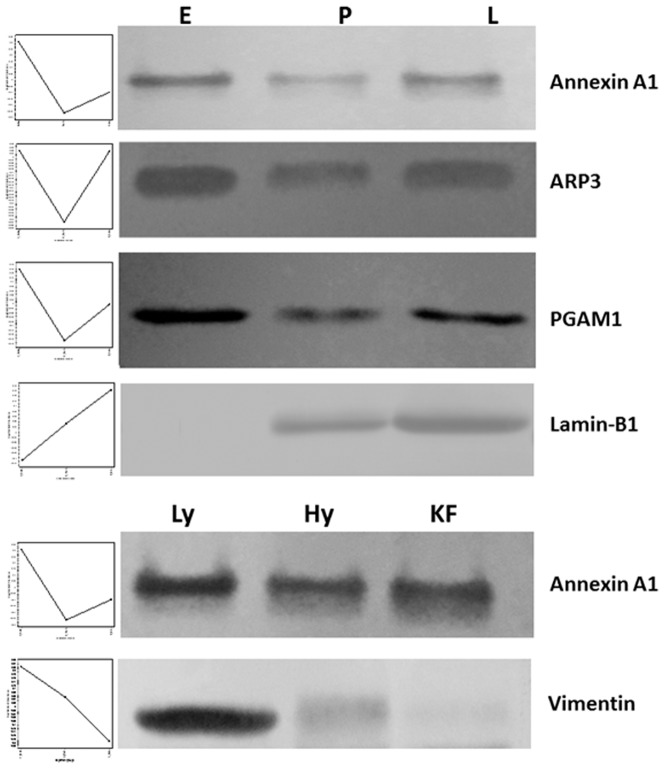
Validation of 2D-DIGE data by western blot. Western blot analysis of annexin A1, ARP3, PGAM1 and Lamin-B1 proteins were differentially regulated during lactation stages and annexin A1 and vimentin were differentially regulated in high and low-milk yielding samples.

**Figure 12:**
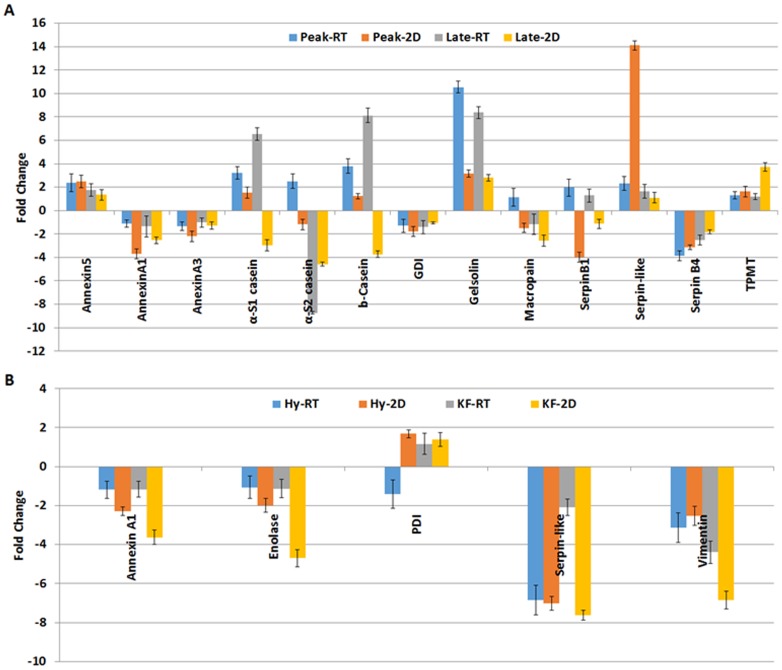
Validation of 2D-DIGE data by quantitative PCR and compared with the 2D-DIGE data. Quantitative PCR analysis was performed for the genes shown in the figure and compared its fold expression change in relation to the 2D-DIGE data. **A.** The expression profile of proteins at peak and late stage of lactation were represented in comparison to early stage that was normalized to 1. **B.** The expression profile of proteins of Hy and KF samples were represented in comparison to Ly samples that was normalized to 1.

The expression levels of some differentially regulated proteins during lactation were confirmed by western blot and quantitative PCR. Antibodies with bovine cross reactivity were not available for most of these proteins and we performed western blot for annexin A1, actin-related protein 3 homolog (ARP3), phosphoglycerate mutase 1 (PGAM1), lamin B1 and vimentin whose antibodies were available commercially and can cross react with bovine. The western blot analysis of these five proteins corresponds to expression profile of proteomics data (Figure 11). We performed quantitative PCR for some of these proteins but the expression at protein levels were not always correlated to mRNA levels (Figure 12). Expression levels of casein isoforms, macropain, serpin B1 and protein disulfide isomerase did not correlate with its mRNA levels. The results indicate that the differentially expressed proteins during lactation stages and milk yielding variability are regulated at protein level and/or due to post-translational modifications of these proteins.

## Conclusions

The dynamic proteome profile of mammary epithelial cells at three different stages of lactation and differential expression of proteins in MEC of high and low milk producing ability was studied using 2D-DIGE and mass spectrometry. We found 41 proteins that are differentially expressed during three stages of lactation and 22 proteins associated with milk yielding. Most of these proteins have binding and catalytic functions that are associated with metabolic processes. The PCA and HC analysis supports the biological variation of expression changes at different stages of lactation. Up-regulated proteins during peak stage and both high yielding samples are mostly associated in increasing milk production; whereas the up-regulated proteins at late stage or low milk yielding are involved in stress induced signaling pathways. Network analysis of the differentially regulated proteins revealed that the up-regulated proteins in late stage connect to the NF-κB and Jnk-MAPK pathways causing decline in milk production. Akt, PI3K and p38/MAPK signaling pathways are associated with high milk production mediated through insulin hormone signaling. At protein level the expression profile were correlated with proteomics data, however at mRNA levels it corresponded with few identified proteins indicating the importance of isoforms and post-translational modifications of proteins in regulation of lactation and milk synthesis.

## Supporting Information

Figure S1 **Graphical representation of differentially expressed spots during early, peak and late stages of lactation.**
(PPTX)

Table S1 **List of primers used in RT-PCR or Real-time PCR.**
(DOC)

Table S2 **List of differentially expressed proteins at different stages of lactation identified by mass spectrometry.**
(XLSX)

Table S3 **List of differentially expressed proteins of high and low milk yielding samples identified by mass spectrometry.**
(XLSX)

Table S4 **List of gene names reported in protein pathways or networks identified during different stages of lactation.**
(XLSX)

Table S5 **List of gene names represented in protein pathways or networks identified in high and low milk yield samples.**
(XLSX)
