# Supplementary material for: Comparative 2D-DIGE Proteomic Analysis of Bovine Mammary Epithelial Cells during Lactation Reveals Protein Signatures for Lactation Persistency and Milk Yield
Source: PLoS One. 2014 Aug 11;9(8):e102515. doi: 10.1371/journal.pone.0102515 (PMC4128602; doi:10.1371/journal.pone.0102515)
Supplement: Figure S1 — Graphical representation of differentially expressed spots during early, peak and late stages of lactation. (PPTX) [file pone.0102515.s001.pptx]

## Slide 1
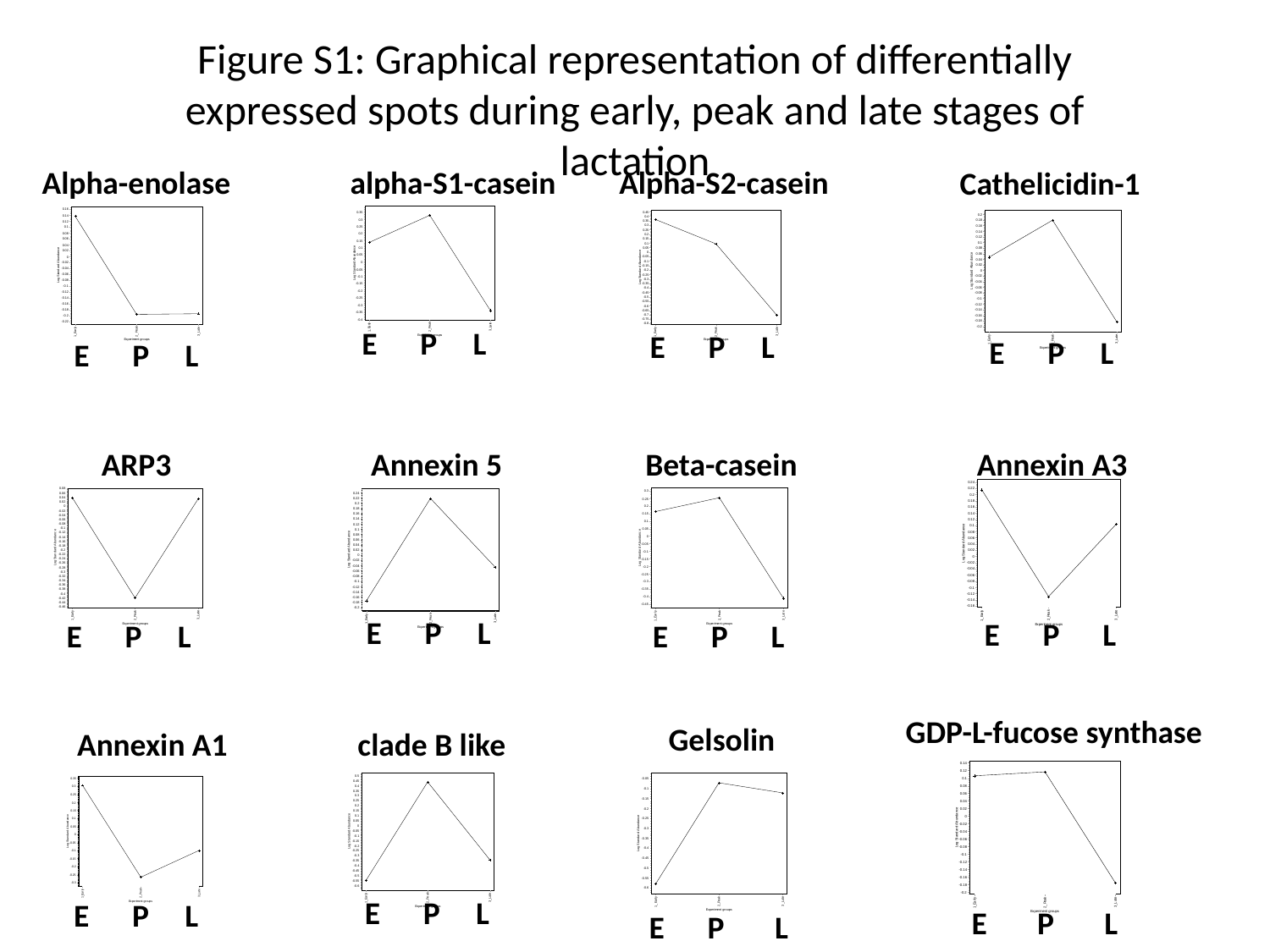

Figure S1: Graphical representation of differentially expressed spots during early, peak and late stages of lactation
Alpha-enolase
alpha-S1-casein
Alpha-S2-casein
Cathelicidin-1
E P L
E P L
E P L
E P L
Annexin A3
Beta-casein
ARP3
Annexin 5
E P L
E P L
E P L
E P L
GDP-L-fucose synthase
Gelsolin
clade B like
Annexin A1
E P L
E P L
E P L
E P L

## Slide 2
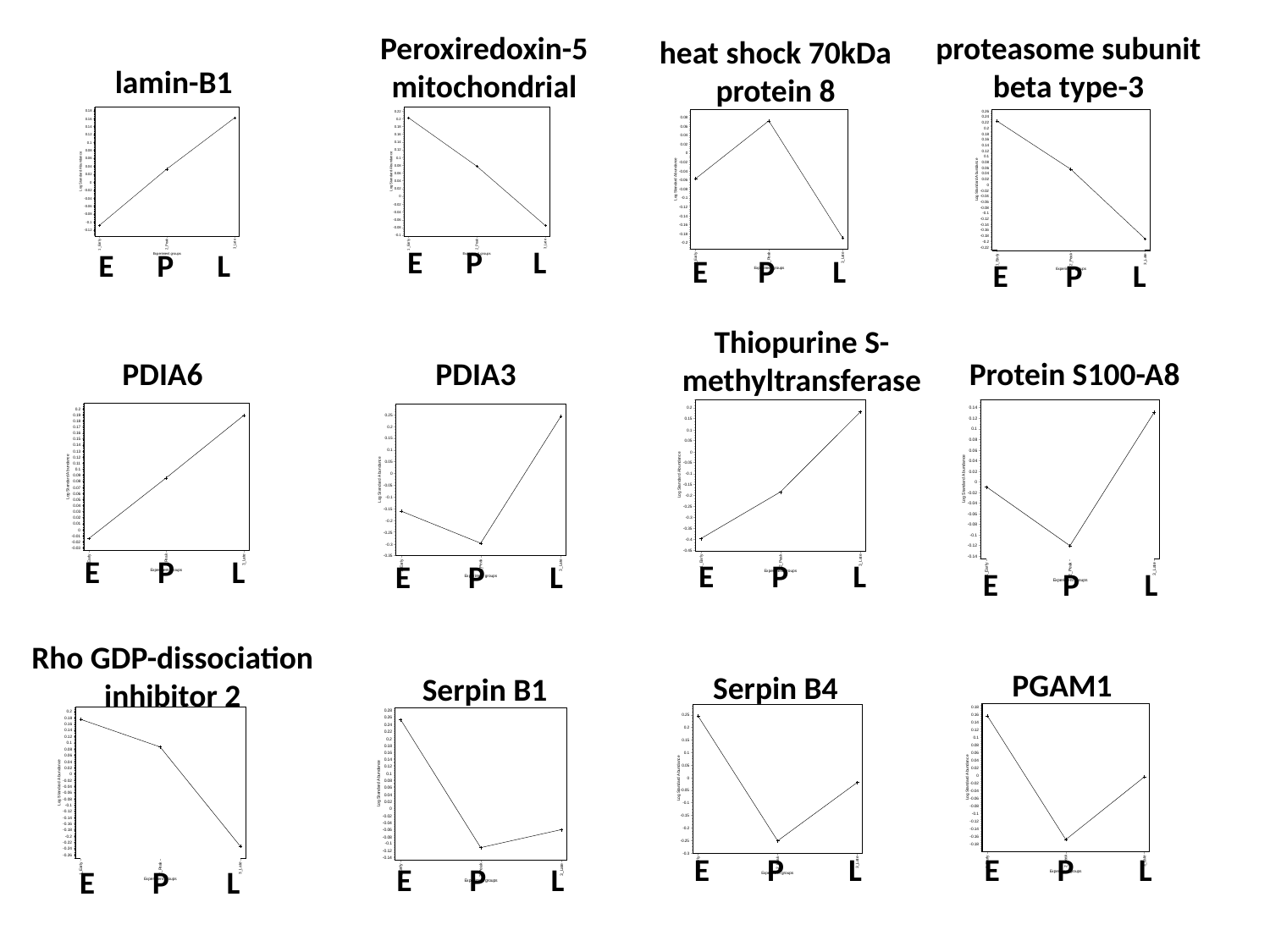

Peroxiredoxin-5
mitochondrial
proteasome subunit beta type-3
heat shock 70kDa protein 8
lamin-B1
E P L
E P L
E P L
E P L
Thiopurine S-methyltransferase
PDIA6
Protein S100-A8
PDIA3
E P L
E P L
E P L
E P L
Rho GDP-dissociation inhibitor 2
PGAM1
Serpin B4
Serpin B1
E P L
E P L
E P L
E P L
