# Supplementary material for: Comparative 2D-DIGE Proteomic Analysis of Bovine Mammary Epithelial Cells during Lactation Reveals Protein Signatures for Lactation Persistency and Milk Yield
Source: PLoS One. 2014 Aug 11;9(8):e102515. doi: 10.1371/journal.pone.0102515 (PMC4128602; doi:10.1371/journal.pone.0102515)
Supplement: Table S1 — List of primers used in RT-PCR or Real-time PCR. (DOC) [file pone.0102515.s002.doc]

**Table S1: List of primers used in RT-PCR or Real-time PCR**

| Gene Name | NCBI Accession Number | Primmer Sequence (5'-3') sense/antisense | Product size (bp) |
| --- | --- | --- | --- |
| Annexin A1 | BC103375 | TTGCTAAGGGTGACCGATCC  ACTCTGCGGAGATGGGGATA | 160 bp |
| Annexin A3 | BC104614 | | CGGCTCCCTAGGTTAGTGTG | | --- |   GCTGTGCATTCGTCCTCTCA | 206 bp |
| Annexin A5 | BT021001 | GAAGATGACTGACGGGAGCC  CAGAAAGGAGCACCGCTACA | 181 bp |
| α-S1 casein | M33123 | CCATGCTCTACATGTCGCCT  TCCCTTTGACTCAGTGGCCT | 213 bp |
| α-S2 casein | DQ173244 | TGCTGTTCCCATTACTCCCA  AGGTTGAGTCCATGGCTTCA | 244bp |
| beta-casein | M16645 | GCTCCTCCTTCACTTCTTGTCC  TGTTTGCTGCTGTTCCTCAC | 219 bp |
| GDI | BC102109 | TTCAACACACCTACCGGACG  CAGGTGAGGTGGTCATGCTT | 190 bp |
| Gelsolin | BC104560 | TTCACTCACAGCCCCACAAG  TCGTCCTGGCTGCATTCATT | 247 bp |
| Macropain | BC123487 | ATCCACACGAGCAGGATGAC  AGAGATACAGCCCCTCTGGG | 223 bp |
| Serpin B1 | BT025443 | TCACCATGGAGCAGCTGAG  GCAGAGCCTTGGACATTTGC | 180 bp |
| Serpin-like | JQ796283 | GGACTTCCAGACCACATCCG  CTTTTGCGGCTCCTCCTCTT | 243 bp |
| Serpin B4 | BC149566 | TGCAGGAGCTTGAAGACCAGCT  CACCCACACCGGTAGCAGCC | 294 bp |
| TPMT | BC118238 | TTGTTTGTGAGCCAGAGCCA  ATACCCGCAGTGCTTTCTCG | 227 bp |
| Enolase | BC103354 | CGGGGCAGATCAAGACTGTT  AAGGAGCCAACGAGTGACTG | 179 bp |
| PDI | M17596 | CCTGCACTCATTATGGCGGA  CGCGCACACAATAGCAATCT | 161 bp |
| Vimentin | AB099035 | ACCCTCTACACCTCGTCCC  TGTTCTGCTGCTCCAGGAAG | 235 bp |
